# Evaluation of the Mechanical and Tribological Behavior of Polyether Ether Ketone Fiber-Reinforced Resin-Based Friction Materials Fabricated by Wet Granulation

**DOI:** 10.3390/polym15244732

**Published:** 2023-12-18

**Authors:** Lekai Li, Zichao Ma, Guoqin Liu, Wei Song, Lili Ren, Shengwang Yuan, Xiao Yang, Qifeng Zhang, Yunhai Ma

**Affiliations:** 1 Key Laboratory of Bionic Engineering (Ministry of Education), College of Biological and Agricultural Engineering, Jilin University, Changchun 130022, China; lilk20@mails.jlu.edu.cn (L.L.); lgq1277993904@163.com (G.L.); liliren@jlu.edu.cn (L.R.); ysw5968@163.com (S.Y.); yangxiao22@jlu.edu.cn (X.Y.);; 2 Institute of Structured and Architected Materials, Liaoning Academy of Materials, Shenyang 110167, China; 3 Weihai Institute for Bionics, Jilin University, Weihai 264200, China; 4 Department of Mechanical Engineering, The Pennsylvania State University, State College, PA 16801, USA; zvm5162@psu.edu; 5 The College of Engineering and Technology, Jilin Agricultural University, Changchun 130022, China; hrsongwei@126.com

**Keywords:** resin-based friction materials, PEEK fiber, wet granulation, tribological properties

## Abstract

Resin-based friction materials (RBFMs) strengthened by polyether ether ketone (PEEK) fiber were designed and prepared in this study. Specimens incorporating PEEK fiber of 2–8 wt.% were fabricated based on wet granulation, and then the effects of the PEEK fiber content on the mechanical and tribological properties of RBFMs were systematically investigated. The results showed that PEEK fiber can sense the braking temperature and then effectively regulate the comprehensive properties of RBFMs. The specimen incorporating 6 wt.% PEEK fiber obtained the optimal comprehensive performance with a stable friction coefficient (COF), excellent fade resistance and recovery properties, and better wear resistance. The worn surface was inspected using a scanning electron microscope. After the friction–wear test, the specimen with 6 wt.% PEEK fiber presented a number of primary and secondary plateaus and a reduced number of pits with wear debris on the worn surface. The study indicated that PEEK fiber could not only enhance the mechanical and tribological properties of RBFMs at low temperatures because of their high strength and self-lubrication but also adhere to wear debris to reduce abrasive wear at high temperatures; furthermore, the adhered wear debris could form a secondary plateau under normal pressure, which could alleviate abrasion.

## 1. Introduction

With the development of technology and the economy, means of transportation such as automobiles and trains are playing a more and more important role in human society, and they have continually increasing requirements in terms of speed, safety and comfort [1]. As an important part in brake systems, resin-based friction materials (RBFMs) are the last line of defense for safe operation; their stability and reliability are directly related to the safety of passengers [2,3]. At present, friction materials applied to brake systems can be divided into four categories: ceramic-based friction materials, resin-based friction materials, powder metallurgy friction materials, and C/C composite materials [4,5,6]. Among them, RBFMs are widely used because of their simple preparation and low cost [4].

**Figure 1:**
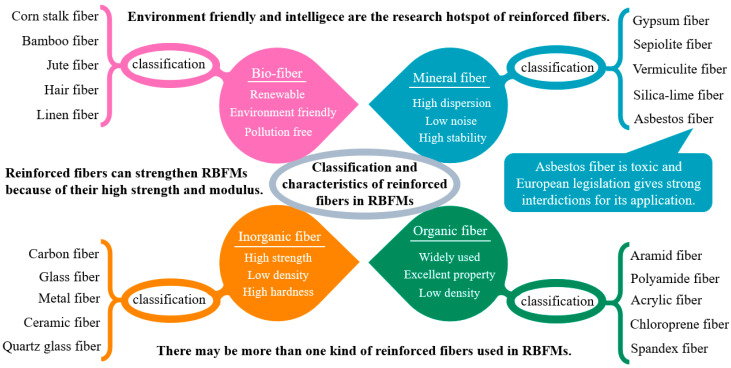
Classification and characteristics of reinforced fibers used in RBFMs.

RBFMs are usually strengthened by reinforced fibers because of their high strength and high modulus [7,8]. Generally speaking, from the macro-perspective, reinforced fibers have a high modulus, high strength, and good thermal and tribological stability, which can be used to enhance strength, fade resistance (the resistance of the friction coefficient (COF) to high-temperature reduction), and wear resistance [9]. From the micro-perspective, reinforced fibers play a positive role in consolidating wear debris, and they are the primary plateau, which can produce nucleation of wear debris, thus promoting the formation of a secondary plateau [10,11,12]. Commonly used reinforced fibers include metal fiber (such as steel fiber and copper fiber, etc.), glass fiber, carbon fiber, ceramic fiber, and organic fiber (including synthetic and natural fibers, such as cotton fiber and corn stalk fiber, etc.) [13,14]. The classification and characteristics of reinforced fibers used in RBFMs are shown in Figure 1.

Reinforced fibers play an important role in RBFMs. Carbon fiber can not only enhance the mechanical strength of RBFMs but also reduce abrasion because of their self-lubrication, high temperature resistance, and other excellent properties (Farhad Ahmadijokani et al.) [12,15,16]. Metal fibers have high strength and high heat conductivity, which can improve the heat conductivity of friction materials, thus preventing COF heat fading during braking (Sung Bin Park, H. Jang et al.) [17,18]; however, because of the environmental pollution caused by copper fiber, they have limited incorporation in RBFMs (Jian Xian, Yang et al.) [19,20]. Natural fibers, which are renewable and non-polluting, have recently become a research hotspot within the field of reinforced fibers (Yunhai Ma [3], Yucheng Liu [21] et al.). Although these fibers can improve tribological properties to a certain extent, they cannot adapt themselves to different braking conditions, and their tribological properties are still poor at higher braking temperatures [22,23].

Polyether ether ketone (PEEK) fiber can reduce abrasion and improve the mechanical strength of RBFMs because of their high strength and self-lubrication [24,25]. Furthermore, PEEK will stay molten at high temperatures (more than 343 °C) and can adhere to wear debris to decrease abrasion [26,27,28]. The adherent wear debris will form a secondary plateau under normal pressure, thus protecting RBFMs and enhancing wear resistance [25]. The characteristics of PEEK fiber provide a good reference for the design of RBFMs. However, the application of PEEK fiber in RBFMs is rarely reported.

Aiming at enhancing the comprehensive properties of RBFMs in different braking conditions, PEEK fiber was incorporated into RBFMs in this study, which responded to the external stimulus with a specific manner and exhibited some desirable behaviors. Firstly, PEEK fiber can enhance the impact strength of RBFMs, which was important for its comprehensive performance. Secondly, molten PEEK can adhere to wear debris on the friction surface at high temperatures, which can alleviate abrasive wear; at the same time, the adhered wear debris can form a secondary plateau under normal pressure to protect RBFMs. Thirdly, the self-lubrication property of PEEK fiber can reduce abrasion and enhance the service life.

## 2. Materials and Methods

### 2.1. Raw Materials

The raw materials in this study mainly included PEEK fiber, sepiolite fiber, compound mineral fibers, phenolic powder, graphite, petroleum coke, aluminum oxide, friction dust (cashew nut shell oil), calcium carbonate, vermiculite powder, barium sulfate, and zinc stearate. The content of barium sulfate decreased step by step with increasing PEEK fiber content because barium sulfate has a smaller effect on the tribological properties of RBFMs. The content of raw materials and their information are shown in Table 1 and Table 2, respectively.

### 2.2. Fabrication of Specimen

**Figure 2:**
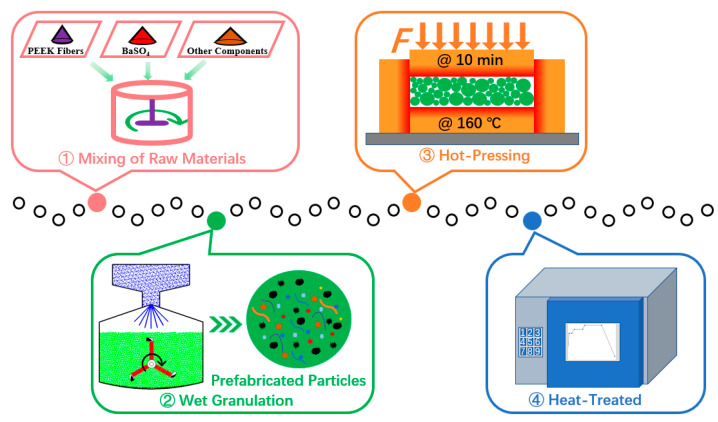
Fabrication process of RBFMs.

The main fabrication steps of RBFMs were step mixing, wet granulation, hot pressing, and heat treatment [21,25]. The fabrication process is shown in Figure 2.

Firstly, the raw materials were mixed using an electrical blender (JF801S, Wangda, Changchun, China). Reinforced fibers such as sepiolite fiber, compound mineral fibers, and PEEK fiber were mixed for 3–5 min to improve dispersion. Then, all other composition were thrown into a compact rake blender (JF810, Wangda, Changchun, China) for mixing, the mixing time was 8–10 min.

**Figure 3:**
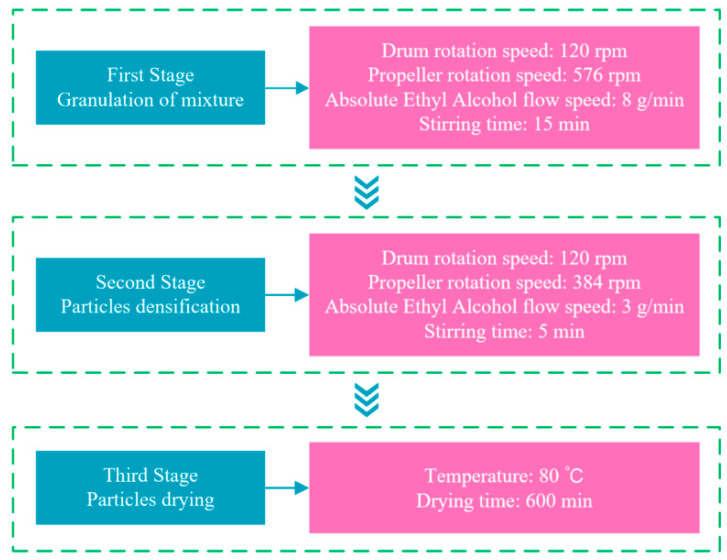
The process of wet granulation.

Secondly, prefabricated particles were prepared by wet granulation using a laboratory tumbling granulator (JF805R, Wangda, Changchun, China). The wet granulation process is presented in Figure 3. The bridging liquid was absolute ethyl alcohol, and the total quantity was about 40 wt.% of the mixture. After wet granulation, the prefabricated particles were dried using a heat-treated case (JF980S, Wangda, Changchun, China).

**Figure 4:**
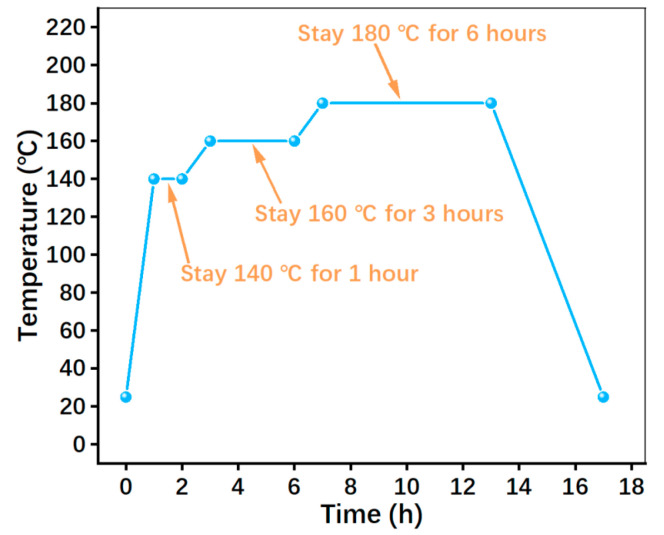
Heat-treatment temperature of specimens.

Thirdly, the prefabricated particles were pressed for 10 min at 160 °C under 45 MPa for hot pressing [25]; the pressing device was a hot compression machine (JFY50, Wangda, Changchun, China). During hot pressing, three intermittent ‘breathings’ were conducted to release volatiles. After hot pressing, the specimens were heat treated by a heat-treated case (JF980S, Wangda, Changchun, China) to remove the remaining stress; the temperature evolution during heat treatment is given in Figure 4.

### 2.3. Testing Methods and Equipment

Thermogravimetric analysis (TGA) was conducted using a TA thermogravimetric analyzer (TGA55, TA, Delaware, USA) under nitrogen, the temperature range from 30 to 400 °C and the heating rate of 5 °C/min. The density of specimens was tested based on an Archimedes Drainage Principle, the hardness of samples was tested by a rockwell hardness measuring instrument (HRSS-150, Hangzhou, China), and the impact strength was tested by an impact tester (XJ-40A, Jinan, Shandong, China). Impact strength was tested according to GB/T 33835-2017; the specimens were cut as 55 mm × 6 mm × 4 mm; the maximum impact energy, swing angle, and impact speed of the pendulum were 0.981 J, 150°, and 2.9 m/s, respectively. Furthermore, the angle of the cutting edge of the striker was 75° and the filleted corner of the cutting edge was 0.8 mm. Each sample was tested five times, and the final results were the average value of the performed 5 tests.

**Figure 5:**
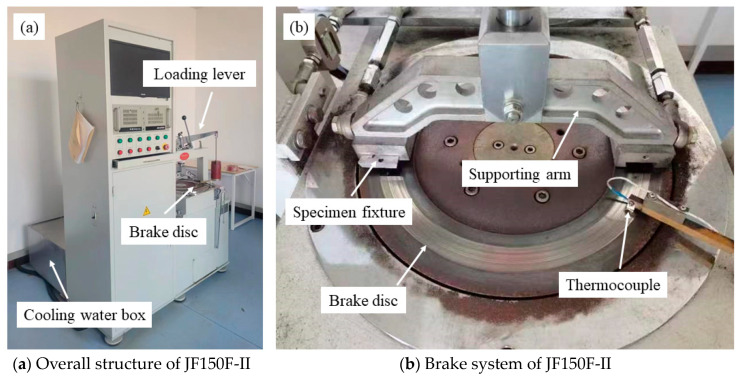
JF150F-II constant-speed tester.

The tribological performance of specimens was evaluated by a constant-speed tester (JF150F-II, Wangda, Changchun, China, shown in Figure 5) according to GB/T 5763-2008. The materials of the brake disc were gray cast iron whose main component was pearlite; the hardness of the brake disc was between 180 and 220 HB; the diameter, thickness, and roughness of the counterpart was 373 mm, 20 mm, and 3.2 μm, respectively. The specimen size of RBFMs was 25 mm × 25 mm × 6 mm; the distance from the specimen center to the brake disc center was 0.15 m. During a friction-wear test, the rotation speed of the brake disc and loading pressure were constant at 480 r/min and 0.98 MPa, respectively. The friction-wear test can be divided into two stages, the first stage was a fade test and the temperatures of the brake disc were set as 100 °C, 150 °C, 200 °C, 250 °C, 300 °C and 350 °C, respectively (the temperature of the brake disc was regulated mainly by cooling water and a thermoelectric couple inside the brake disc. The cooling water and thermoelectric were controlled by the computer). Before the formal test, specimens were run below 100 °C until the contact area reached more than 95%, and then the initial thickness of the specimen was measured with a spiral micrometer. Each sample was measured at 5 different points (including 4 corner points and 1 central point) and they were entered into the computer before the formal test was carried out. During the fade test, the brake disc would rotate for 5000 r and would automatically stop at each temperature; then, the specimen thickness was measured again and input into the computer to automatically calculate COF and SWR according to formulas (1) and (2). The second stage was a recovery test. In this stage, the temperatures was reduced from 300 to 250 °C, 200 °C, 150 °C and 100 °C successively. At each temperature, the brake disc rotated 1500 r until the friction disc automatically stopped. After continuous rotation of 7500 r, the computer would output COF at each temperature. In order to reduce the test errors, 5 parallel tests were carried out for each specimen, and the average value was taken as the test result.

*μ* was defined as COF using Equation (1), SWR of volume loss of Δ*V* was calculated according to Equation (2).

(1) $$\mu =\frac{f}{F_{N}}$$

(2) $$∆V=\frac{1}{2\cdot \pi \cdot r}\cdot \frac{A}{N}\cdot \frac{d_{1}-d_{2}}{f}$$

where *f* was the friction force between the specimen and brake disc (N); *F_N_* was the force generated by normal pressure on the surface (N); *r* was the distance from the specimen center to the brake disc center (*r* = 0.15 m); *n* was the number of revolutions (*n* = 5000); *A* was the contact surface area, which was 625 mm^2^; *d_1_* and *d_2_* were the initial and final thickness of the specimen (mm), respectively.

Due to the frequent or hard braking, the reduction in COF caused by the increasing interface temperature was known as fade; after the release of brakes and cooling down, the extent of revival of the original magnitude of COF was referred to as recovery [29,30]. The fade ratio (*F_fade_*) and recovery ratio (*F_recovery_*) were calculated by Equation (3) and Equation (4), respectively.

(3) $$F_{fade}=\frac{\mu_{F100\;{}^{\circ}C}-\mu_{F350\;{}^{\circ}C}}{\mu_{F100\;{}^{\circ}C}}\cdot 100\%$$

(4) $$F_{recovery}=\frac{\mu_{R100}}{\mu_{F100}}\cdot 100\%$$

where *μ_F_*_100 °C_ was COF at 100 °C during the fade test, *μ_F_*_350 °C_ was COF at 350 °C during the fade test, and *μ_R_*_100 °C_ was COF at 100 °C during the recovery test.

After the friction-wear test, the worn surface morphology was evaluated by a scanning electron microscope (SEM, EVO-18, ZEISS, Jena, Germany). The samples for SEM tests were carefully cut into 5 mm × 5 mm sections perpendicular to worn surface using a cutterbar (JF151F, Wangda, Changchun, China), which would not destroy the worn surface morphology, and a thin layer of gold dust was coated on the worn surface using a carbon coater (SBC-12, KYKY Technology, Beijing, China) to ensure the conductivity [31].

## 3. Results and Discussion

### 3.1. Thermogravimetric Analysis

**Figure 6:**
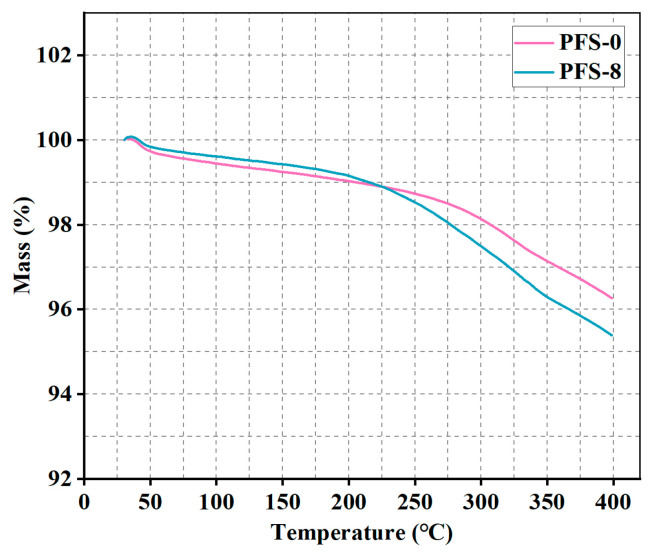
TGA curve of PFS-0 and PFS-8.

The thermographs of PFS-0 and PFS-8 are shown in Figure 6. It can be inferred that PFS-0 and PFS-8 showed the first weight loss below 100 °C, which can be attributed to the water evaporation or dehydration of water molecules in the samples [32]. Between 100 and 225 °C, the weight loss of PFS-0 was higher than that of PFS-8; with temperature continuously increasing (from 225 to 400 °C), the weight loss of PFS-8 was higher than that of PFS-0, which was mainly caused by the cross-linking reaction between the PEEK fiber and phenolic resin.

### 3.2. Density and Hardness

**Figure 7:**
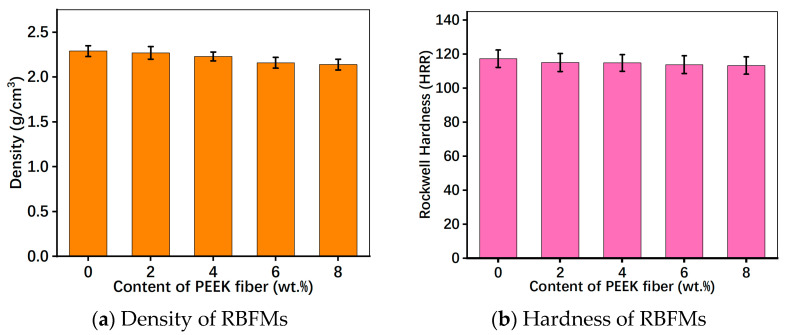
Density and hardness of RBFMs strengthened by PEEK fiber.

Figure 7a presents the density of RBFMs strengthened by PEEK fiber. It can be observed that the density of specimens decreased with the increasing PEEK fiber content, which could be ascribed to the replacement of low-density PEEK fiber with high-density barium sulfate. Among all samples, PFS-0 had the maximum density and PFS-8 had the minimum density.

Figure 7b shows the hardness of RBFMs strengthened by PEEK fiber. It can be inferred that with the increasing content of PEEK fiber, hardness showed a decreasing trend, which might be caused by the low hardness of the PEEK fiber. Samples presented a hardness in the range of 113.3–117.3 HRR. Furthermore, on the whole, the hardness of specimens with different PEEK fiber content changed slightly.

### 3.3. Impact Strength

**Figure 8:**
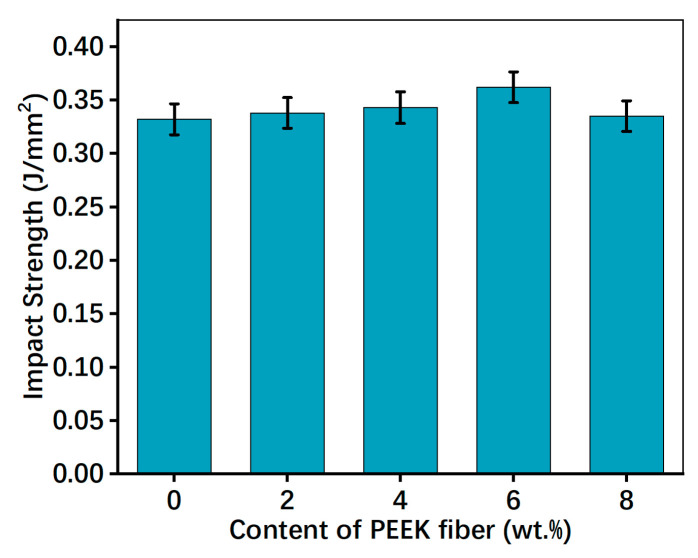
Impact strength of RBFMs strengthened by PEEK fiber.

Impact strength is the impact resistance of friction materials, which can be used to evaluate the toughness and brittleness [15]. To confirm the influence of PEEK fiber on mechanical properties of RBFMs, the impact strength was evaluated by an impact tester. Figure 8 presents the impact strength of RBFMs with different PEEK fiber content. It could be inferred that the PEEK fiber could enhance the impact strength of RBFMs. The impact strength showed an increasing at first and then a decreasing trend with increasing PEEK fiber content. PFS-6 had the maximum impact strength among all samples (0.362 J/mm^2^), which was 9.04% higher than that of PFS-0. The enhanced impact strength could be attributed to the bearing capacity of PEEK fiber [15]; during the impact process, the PEEK fiber can not only bear the stress in RBFMs but also transfer and disperse the stress more effectively, thus showing an improved impact strength [7,17].

### 3.4. Tribological Properties

**Figure 9:**
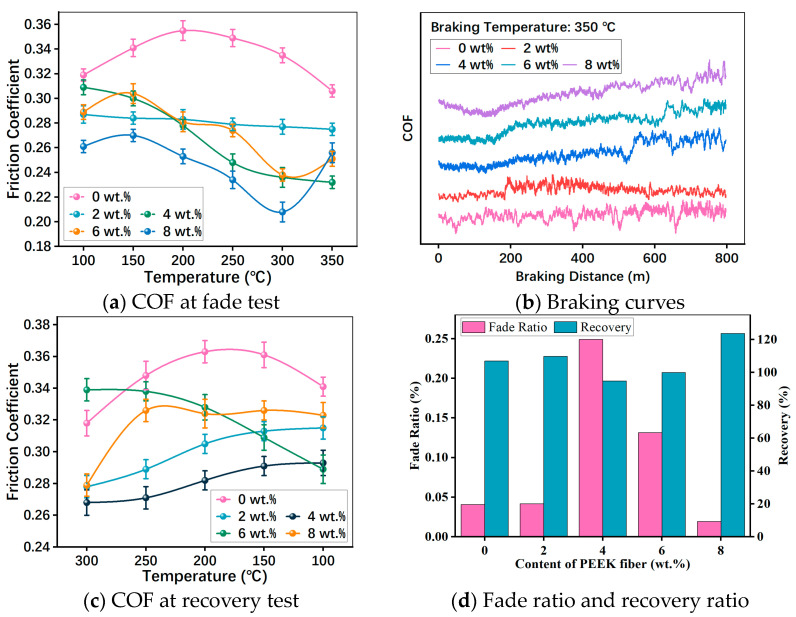
COF of RBFMs strengthened by PEEK fiber.

Figure 9 presents the COF of RBFMs with different amounts of PEEK fiber at the normal pressure and sliding velocity of 0.98 MPa and 7.536 m/s, respectively. As seen in Figure 9a, the introduction of PEEK fiber caused a reduction in COF. At different braking temperatures, samples holding PEEK fiber exhibited a smaller COF. Indeed, the high strength, high modulus and the formation of immobilized interfacial zone around the PEEK fiber can cause the dissipation of a major portion of braking stress exerted during braking, thus reducing the COF [15]. Moreover, the introduction of PEEK fiber can not only enhance the mechanical properties of RBFMs but also reduce COF and SWR because of its self-lubricating property [33]. During the fade test, PFS-2 showed the slightest COF variation among all samples with increasing braking temperatures, which obtained the optimal COF stability. Furthermore, Figure 9b shows the braking curves at 350 °C; it can be inferred in Figure 9b that PFS-2 had the most stable braking process at the same temperature (350 °C), where there was no obvious braking instability.

As revealed by Figure 9a, the COF of PFS-0 (with PEEK fiber of 0 wt.%) experienced an increase at first and then a decreasing trend with the increase in temperature. This is consistent with the previous results [21]. A possible explanation of the increasing COF between 100 and 200 °C was that the exposed fibers (such as PEEK fiber, sepiolite fiber, and compound mineral fibers) on the friction surface would scrape the brake disc, which was transformed into friction output [34]. With the further increasing in braking temperature, the COF begun to reduce, which could be attributed to the decreasing shear strength of phenolic resin at higher temperatures [2]. In addition, the lubrication components from the degradation of phenolic resin at high temperatures could also cause a decreased COF [3]. Interestingly, the behaviors of samples holding PEEK fiber were quite different. Specifically, specimens having less PEEK fiber (2 wt.% and 4 wt.%) reported a COF decreasing trend with the increasing temperature, while specimens holding more PEEK fiber (6 wt.% and 8 wt.%) showed a COF variation trend of increasing from 100 to 150 °C, dropping from 150 to 300 °C and increasing again from 300 to 350 °C (shown in Figure 9a).

Figure 9c shows the COF in the recovery test. It served to show that the self-lubrication property of the PEEK fiber could still reduce COF with decreasing temperatures. The COF of PFS-0 (incorporating PEEK fiber of 0 wt.%) kept a higher COF at different braking temperatures. The COF of PFS-0 exhibited an increase at first and then a decreasing trend with reducing braking temperatures; the COF of PFS-2 and PFS-4 kept increasing with decreasing braking temperatures; whereas PFS-6 had the opposite trend with that of PFS-2 and PFS-4; finally, PFS-8 showed a COF variation trend of increasing firstly and then remained stable.

To further evaluate the COF variation, it made sense to determine the fade ratio and recovery ratio. As evident from Figure 9d, the fade ratio order was PFS-4 > PFS-6 > PFS-2 > PFS-0 > PFS-8; the result showed that PFS-8 (incorporating PEEK fiber of 8 wt.%) had the optimal fade ratio (1.92%), which had a better fade resistance than that of Farhad Ahmadijokani [12] and Amar Patnaik [35] reported. As illustrated in Figure 9d, the recovery ratio order was PFS-8 > PFS-2 > PFS-0 > PFS-6 > PFS-4, and PFS-6 obtained the first-rank recovery property (100%). Interestingly, PFS-2 also had a better recovery ratio of 113.94%.

Summing up the above, the incorporation of PEEK fiber could improve tribological properties of RBFMs. Comparing samples holding different content of PEEK fiber, PFS-2 had the more outstanding fade resistance and recovery property, and it had more pronounced friction stability during the whole test.

**Figure 10:**
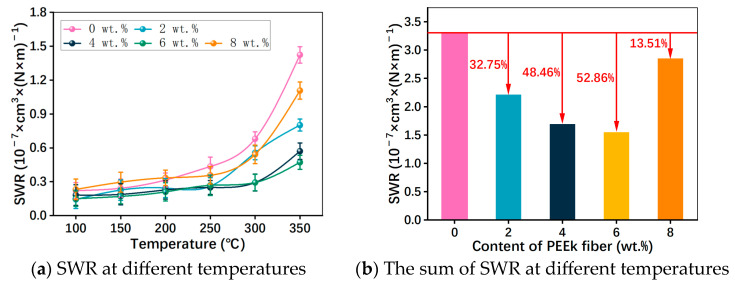
SWR of RBFMs strengthened by PEEK fiber.

The composition and fabrication process can influence the wear resistance of RBFMs, which finally determines the service life [36]. In this study, the wear resistance of RBFMs incorporating different PEEK fiber content was evaluated by comparing SWR at different braking temperatures and the sum of SWR. Figure 10a displayed the SWR variation trend at different braking temperatures. The friction-wear test demonstrated that SWR of RBFMs was remarkable affected by PEEK fiber; the SWR of samples holding PEEK fiber was lower than that of PFS-0 especially at high temperatures; moreover, it increased with increasing braking temperature. Our results showed similar trends with the reported work of Yucheng Liu [21]. SWR observably increased with increasing temperature from 250 to 350 °C. In this temperature range, the bonding strength between phenolic resin binder and other reinforcement composition was weakened, which resulted from the thermal degradation of phenolic resin matrix [37,38], thus significantly reducing the shearing strength [2]. In addition, the glass transition of PEEK fiber also occurred in this temperature range [25], which would also cause an increscent SWR. Figure 10b showed the sum of SWR at each temperature. It could be inferred that the incorporation of PEEK fiber can reduce the sum of SWR by 13.51%–52.86% and PFS-6 had the optimal wear resistance. Furthermore, the wear resistance of RBFMs strengthened by PEEK fiber was higher than that of RBFMs strengthened by glass fiber [39], jute fiber [40] and corn stalk fiber [21].

In conclusion, the promoted wear resistance of RBFMs by PEEK fiber could be ascribed to the bearing function at lower temperatures and the adhesion function of wear debris at high temperatures to facilitate the formation of secondary plateau [25]. Additionally, the PEEK fiber can form a layer of lubrication film because of its self-lubrication properties, which can also reduce abrasion [27].

### 3.5. Wear Mechanisms

Primary and secondary plateaus, adhesive pits, wear debris and grooves on worn surface can be used to analyze the wear mechanisms of friction materials [41]. The worn surface morphology was characterized by SEM to analyze wear mechanisms, thus obtaining the mechanism for the improved wear resistance by PEEK fiber.

**Figure 11:**
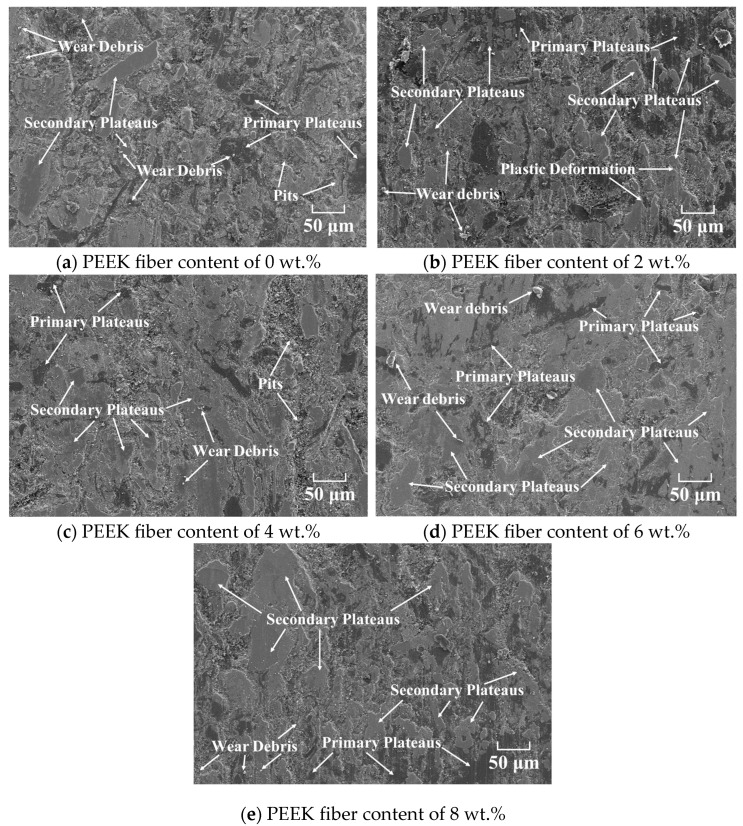
Worn surface morphology of RBFMs strengthened by PEEK fiber.

As observed in Figure 11a, a large amount of wear debris and pits were found, indicating that the wear mechanisms were severe abrasive wear and adhesive wear [5]. In fact, the composition of RBFMs can determine wear resistance and then influence its wear mechanisms [38]. The serious abrasion of samples incorporating no PEEK fiber can be attributed to the lack of strengthening effect and self-lubrication film by PEEK fiber [21,42].

**Figure 12:**
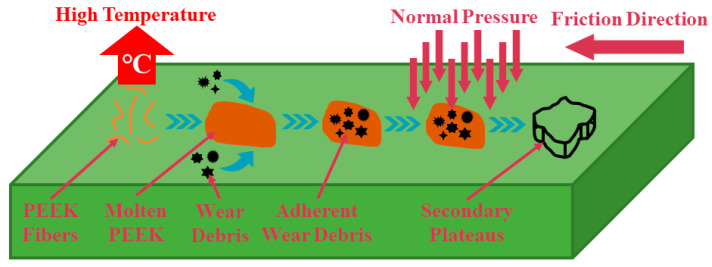
Wear mechanism of RBFMs strengthened by PEEK fiber at high temperatures.

The PEEK fiber in RBFMs mainly played a bearing and self-lubrication role at lower braking temperatures. While the braking temperature was more than its melting point of 343 °C, the molten PEEK fiber would adhere to wear debris on a worn surface to reduce abrasive wear; and under the action of normal pressure, the adhered wear debris would form a secondary plateau, which can protect friction materials from being hurt by wear debris and the brake disc (shown in Figure 12). Figure 11b–e present the worn surface morphology of RBFMs incorporating different content of PEEK fiber. It served to show that the wear mechanisms of RBFMs holding less PEEK fiber (2 wt.%) were mainly abrasive wear and adhesive wear; there were some wear debris, plastic deformation and little primary and secondary plateaus on worn surface. It also served to show in Figure 11b that a lower content of PEEK fiber cannot achieve effective adhesion to wear debris; thus, it presented a relatively rough worn surface.

With increasing PEEK fiber content (4–6 wt.%), a large amount of wear debris was adhered by molten PEEK and was compacted as the secondary plateau, which was manifested as a more smooth worn surface (shown in Figure 11c,d). There was a little amount of wear debris and pits on the worn surface; meanwhile, a large amount of primary and secondary plateaus could be observed. Generally speaking, the primary plateau was composed of components with higher mechanical strength and better wear resistance (such as reinforced fibers and hard particles) [34,43], and these primary plateaus acted as a barrier, reducing the movement of wear debris, making them tend to stop and stick together on the worn surface and be compacted, thus promoting the formation of a secondary plateau [44,45,46]. Many studies have shown that the formation of a secondary plateau on worn surfaces played an important role in improving tribological properties [39,47]. With the PEEK fiber content continuously increasing (8 wt.%), the thermoplastic PEEK and thermosetting phenolic resin were cross-linked and cured under high temperatures, which caused a reduction in wear resistance, showing a more rough worn surface. This was in agreement with our previous study [25].

## 4. Conclusions

To overcome the disadvantages of lacking adaptive capacity to different braking conditions of traditional RBFMs, a new RBFM strengthened by PEEK fiber was developed, which provided a novel idea for the research of intelligent friction materials. The mechanical and tribological properties can be effectively regulated by the content of PEEK fiber. The impact strength and tribological properties were not significantly improved with a lower content of PEEK fiber (2 wt.%). With the increase in PEEK fiber content (4 wt.%), the impact strength and wear resistance increased due to the high strength and self-lubrication of PEEK fiber; however, the fade resistance decreased. As the PEEK fiber content reached to 6 wt.%, samples showed the optimal comprehensive performances. With PEEK fiber continuously increasing (8 wt.%), its comprehensive properties began to decline to a certain extent. Furthermore, chemical enhancement on PEEK fiber might be also worthwhile for enhancing the comprehensive performances of RBFMs [48], and we will investigate the influences of chemical treatment for PEEK fiber on dielectric, thermal, mechanical and tribological properties of RBFMs in the future.

## Figures and Tables

**Table 1:** Composition of the prepared specimens.

Raw Materials (by wt.%) and Size (by Mesh)	Specimens				
	PFS-0	PFS-2	PFS-4	PFS-6	PFS-8
PEEK fiber (0.1 mm× 3 mm)	0	2.00	4.00	6.00	8.00
Sepiolite fiber (0.15 mm × 2.5 mm)	5.00	5.00	5.00	5.00	5.00
Compound mineral fibers (0.2 mm × 3 mm)	20.00	20.00	20.00	20.00	20.00
Phenolic powder (200)	9.00	9.00	9.00	9.00	9.00
Graphite (100)	8.00	8.00	8.00	8.00	8.00
Petroleum coke (400)	7.00	7.00	7.00	7.00	7.00
Aluminum oxide (325)	6.00	6.00	6.00	6.00	6.00
Friction dust (cashew nut shell oil, 100)	2.00	2.00	2.00	2.00	2.00
Calcium carbonate (1250)	13.00	13.00	13.00	13.00	13.00
Vermiculite powder (30)	5.00	5.00	5.00	5.00	5.00
Barium sulfate (325)	24.00	22.00	20.00	18.00	16.00
Zinc stearate (200)	1.00	1.00	1.00	1.00	1.00

**Table 2:** The information of raw materials.

Raw Materials	Raw Materials Information			
	Purity		Supply	Function
PEEK fiber			Changzhou Chuangying New Material Technology Co., LTD, Changzhou, China	Reinforce mechanical strength, adhering wear debris on worn surface
	
Sepiolite fiber	99%		Lingshou Jiasuo Building Materials Processing Co., LTD, Shijiazhuang, China	Reinforce mechanical strength
Compound mineral fibers	SiO_2_: 40–43%; Al_2_O_3_:16–18%; CaO: 14–16%; MgO: 5–7%; Fe_2_O_3_: 3–5%; C: 4–6%		Shijiazhuang Mayue Building Materials Co., LTD, Shijiazhuang, China	Reinforce mechanical strength
Phenolic powder			Henan Borun Casting Material Co., LTD, Gongyi, China	Adhere to the other reinforcement components
	
Graphite	99.5%		Henan Borun Casting Material Co., LTD, Gongyi, China	Form friction film to enhance tribological properties
Petroleum coke	85%		Shijiazhuang Yuxin Building Materials Co., LTD, Shijiazhuang, China	Reduce COF and specific wear rate
Aluminum oxide	99.9%		Henan Borun Casting Material Co., LTD, Gongyi, China	Reduce adhesive wear, enhance COF and wear resistance
Friction dust (cashew nut shell oil)			Zhejiang Jiamin Plastic Co., LTD, Jiaxing, China	Improve braking stability and reduce braking noisy
	
Calcium carbonate	99%		Shandong Yusuo Chemical Technology Co., LTD, Linyi, China	Filler in RBFMs
Vermiculite powder			Lingshou Xuyang Mining Co., LTD, Shijiazhuang, China	Reduce braking noise and density of RBFMs
	
Barium sulfate	≥98%		Shandong Yusuo Chemical Technology Co., LTD, Linyi, China	Reduce braking noise
Zinc stearate	Zinc content: 11.01%; Free acid content: 0.47%; Moisture content: 0.37%		Wuxi Yatai Joint Chemical Co., LTD, Wuxi, China	Lubrication

## Data Availability

Data are contained within the article.
